# Effects of a high salt diet on blood pressure dipping and the implications on hypertension

**DOI:** 10.3389/fnins.2023.1212208

**Published:** 2023-07-03

**Authors:** Jesse Viggiano, Dominic Coutinho, Maya N. Clark-Cutaia, Diana Martinez

**Affiliations:** ^1^Department of Biomedical Sciences, Cooper Medical School of Rowan University, Camden, NJ, United States; ^2^NYU Rory Meyers College of Nursing, New York, NY, United States

**Keywords:** hypertension, neural control, circadian blood pressure, baroreflex, nucleus of solitary tract

## Abstract

High blood pressure, also known as hypertension, is a major risk factor for cardiovascular disease. Salt intake has been shown to have a significant impact on BP, but the mechanisms by which it influences the blood pressure dipping pattern, and 24-h blood pressure remains controversial. This literature review aims to both summarize the current evidence on high salt diet induced hypertension and discuss the epidemiological aspects including socioeconomic issues in the United States and abroad. Our review indicates that a high salt diet is associated with a blunted nocturnal blood pressure dipping pattern, which is characterized by a reduced decrease in blood pressure during the nighttime hours. The mechanisms by which high salt intake affects blood pressure dipping patterns are not fully understood, but it is suggested that it may be related to changes in the sympathetic nervous system. Further, we looked at the association between major blood pressure and circadian rhythm regulatory centers in the brain, including the paraventricular nucleus (PVN), suprachiasmatic nucleus (SCN) and nucleus tractus solitarius (nTS). We also discuss the underlying social and economic issues in the United States and around the world. In conclusion, the evidence suggests that a high salt diet is associated with a blunted, non-dipping, or reverse dipping blood pressure pattern, which has been shown to increase the risk of cardiovascular disease. Further research is needed to better understand the underlying mechanisms by which high salt intake influences changes within the central nervous system.

## Introduction

Across the United States, cardiovascular disease (CVD) including hypertension is the leading cause of death amongst men and women, and almost all ethnic groups. In 2020, CVD claimed the lives of 697,000 individuals; 1 in 5 deaths were attributable to CVD (Centers for Disease Control and Prevention, National Center for Health Statistics, 2022). Conditions such as coronary artery disease, arrhythmias, heart failure, and cardiac valve disease drastically alter the lives of those who acquire them: physically, emotionally, and financially. Between 2016 and 2017, CVD cost the United States approximately $229 Billion in healthcare services, prescription medications, and lost productivity due to illness and death (Agency for Healthcare Research and Quality, 2021). As of 2022, about 20.1 million adults over the age of 20 have been diagnosed with coronary artery disease, translating into approximately 805,000 heart attacks per year (Tsao et al., 2022). From 1997 to 2019, the prevalence of heart disease in individuals 18 years and older has increased from 5.9 to 6.4% (N.C.f.H. Statistics, 2022). But what is causing this increasing incidence of CVD across our country? Although the answer to this is multifaceted, one answer lies in the American diet.

As our nation quickly progressed into a fast paced, work around the clock society, Americans have begun resorting to convenient food options that are packed with sodium, trans fats, and other unhealthy, unnatural substances (Lee et al., 2022). Despite recent evidence of the health issues associated with over consumption of processed foods, the rate of consumption has not changed in the last 18 years (Zeng et al., 2019). Additionally, 53.6 million Americans live more than a half-mile away from reliable food sources in urban areas, or further than 10 miles in a rural area (USDA, 2022), making access to healthy food options limited in a vast number of communities across the country. Amongst the various health implications that come with an unhealthy high-sodium diet, the most concerning is hypertension.

According to the American Medical Association, hypertension is an abnormally increased arterial blood pressure, greater than 130/80 mmHg. Some causes of hypertension include a diet with processed foods with salt, fat, and cholesterol (Whelton et al., 2017). Among hypertensive individuals in America, 86% were found to be consuming more than the recommended 2,300 mg of sodium per day (Jackson et al., 2016). Processed foods have excess sodium for various purposes, such as palatability, shelf stability, moisture retention in meat products, and prevention of bacterial growth in cheeses (Haron et al., 2020). Approximately 116 million U.S. adults are living with hypertension currently, making up roughly 47% of the at-risk population (Ostchega et al., 2020). There are also millions of Americans that have undiagnosed hypertension, therefore, reported statistics are grossly underestimated. One study showed that in a review of 126,699 patient records, 37.3% had undiagnosed hypertension, and 27% were diagnosed but never prescribed an anti-hypertensive medication (Huguet et al., 2021).

Hypertension is not only an issue in the United States, however. Shockingly, the United States does not even fall within the top 10 countries with the highest prevalence of hypertension. According to Zhou et al., prevalence of hypertension in men reached as high as 62% in Paraguay and 56% in Hungary in 2019 (NCD Risk Factor Collaboration (NCD-RisC), 2021). About 54% of strokes and 47% of coronary heart disease, worldwide, are attributable to hypertension (Wu et al., 2015). An observational study conducted by Campagnoli et al. (2012) found that university students in Asuncion, Paraguay had significantly increased salt intake through 24-h urine samples (Campagnoli et al., 2012). Further, they had a higher blood pressure versus their peers in other countries. Daily sodium consumption in many countries was much higher than the WHO suggested amount (Table 1). The increase in hypertension may differ between high and low-income countries (Schutte et al., 2021). Thus, understanding the role of a high salt diet in the development of hypertension is a global concern (Schutte et al., 2021).

**Table 1:** Country distribution of daily sodium consumption.

Country	Intake (g/day)		Sources
	Male	Female	
Brazil	4.3	3.9	Powles et al. (2013)
China	8.8–17.2	7.5–14.6	Liu (2009)
Malaysia	3.5	2.8	Abdul Aziz et al. (2021)
South Korea	3.8	2.7	Kim et al. (2022)
Iran	9.0–11.8	8.4–10.6	Pourkhajoei et al. (2022)
United States	3.5	2.7	Lennie et al. (2020)

Although the physiological mechanisms underlying hypertension are still not completely understood, it is widely accepted that a high salt diet greatly influences the body’s ability to regulate blood volume due to electrolyte imbalances (Weinberger, 1996). In this review, we will discuss the mechanisms by which this occurs and how a chronic high salt diet may lead to neurogenic hypertension. Neurogenic hypertension describes the phenomenon in which a patient’s elevated blood pressure is mediated by an elevated sympathetic nervous system activity (Mann, 2018). We will review various studies on animal models and how a high salt diet influences various brain regions specialized to regulate blood pressure, and how these findings are reflected in humans. Lastly, we will explore the effects of a high salt diet on circadian blood pressure control and discuss improving overall health to combat the increasing incidence of hypertension.

## Baroreceptor modulation of acute changes in blood pressure

At the most basic level, it is well understood that the consumption of a high salt diet alters the body’s homeostatic electrolyte balance, specifically the amount of extracellular sodium in circulation which in turn alters the body’s ability to clear extra circulatory volume (Denton et al., 1995; Robinson et al., 2019). Within the cardiovascular system, fluid shifts to areas with a higher osmotic gradient, largely influenced by sodium. With this general principle in mind, increasing the amount of sodium in the circulating blood draws water out of the surrounding tissue, and into the bloodstream to balance the hypernatremia. The net effect of this is a transient increase in blood pressure solely due to increased blood volume, and the heart being forced to move more blood with each beat (Intersalt Cooperative Research Group, 1988). To correct for this, the body has highly specialized nerve endings embedded within the aortic arch and bilateral carotid sinuses, called “baroreceptors,” which modulate these transient spikes in blood pressure. In response to an increase in intravascular pressure, baroreceptors increase their firing rate to the nucleus of the solitary tract (nTS), inhibiting its sympathetic output to the heart and vasculature. This causes a decrease in heart rate via decreased sympathetic output to the Sinoatrial (SA) node in the heart, as well as vasodilation. Additionally, the nTS sends direct excitatory projections to the nucleus ambiguous to enhance parasympathetic output and reduce heart rate (Stuesse and Fish, 1984; Machado and Brody, 1988). In combination, these resulting actions lead to a decrease in the mean arterial pressure (Armstrong et al., 2023).

The Baroreflex feedback pathway is the body’s primary means by which it regulates small fluctuations in blood pressure. Studies suggest that these baroreceptors have the capacity to regulate blood pressure even in patients with chronic hypertension through a mechanism termed “resetting,” allowing them to modulate changes in blood pressure over a wide range of pressures, rather than pressures confined to a certain range (McCubbin et al., 1956). Due to its important role in regulation of blood pressure, the nTS has been the focus of much research with respect to high salt diets.

Baroreflex dysfunction is associated with hypertension in humans, and in various models of diet-induced hypertension including the Dahl-Salt Sensitive (DSS) rat, deoxycorticosterone acetate (DOCA)-salt and spontaneously hypertensive rat (SHR). Gordon et al. (1981) showed differences in baroreflex sensitivity in salt sensitive rats exposed to high salt diets versus those given a low salt diet (Gordon et al., 1981). However, they determined that since the two groups of rats had statistically similar arterial blood pressures, arterial distensibility could not be the driving factor mediating the difference in sensitivity. They theorized that the root of the dysfunction was neuronal. But they did not specify the exact central mechanism. Miyajima and Bunag (1987) found that dysfunction in sympathetic attenuation in Dahl-Salt Sensitive rats given a high salt diet lead to blunted sympathetic withdrawal at higher arterial blood pressures, making the baroreceptor reflex less effective (Miyajima and Bunag, 1987). A follow-up study suggested that this defect is the result of elevations in the baroreceptor pressure threshold that triggers the afferent fibers to send inhibitory signals to the nTS to block the sympathetic output of the baroreflex (Andresen, 1989).

Other rat models also displayed similar disruptions in baroreceptor activity in response to high salt diets. In SHR, salt loading similarly lead to accelerated hypertension as well as increased renal sympathetic nerve activity and impaired central baroreceptor reflex activity (Ono et al., 1997). However, the young normotensive Wistar-Kyoto rats (WKY) used as their control group displayed facilitation of the baroreceptor reflex without significant changes in renal sympathetic nerve activity or arterial pressure in response to the same salt loading (Ono et al., 1997). Takeda et al. (1988) aimed to determine whether the central changes in baroreflex preceded the onset of clinical hypertension in DOCA-salt treated rats (Takeda et al., 1988). Compared to control rats treated with saline injection and a normal salt diet, they found that DOCA-salt treated rats displayed significantly decreased bradycardic, vasodepressor, and sympatho-inhibitory responses following direct stimulation of the aortic depressor nerve which served as the trigger for the onset of the baroreflex.

## Circadian blood pressure disruption is associated with hypertension

**Figure 1:**
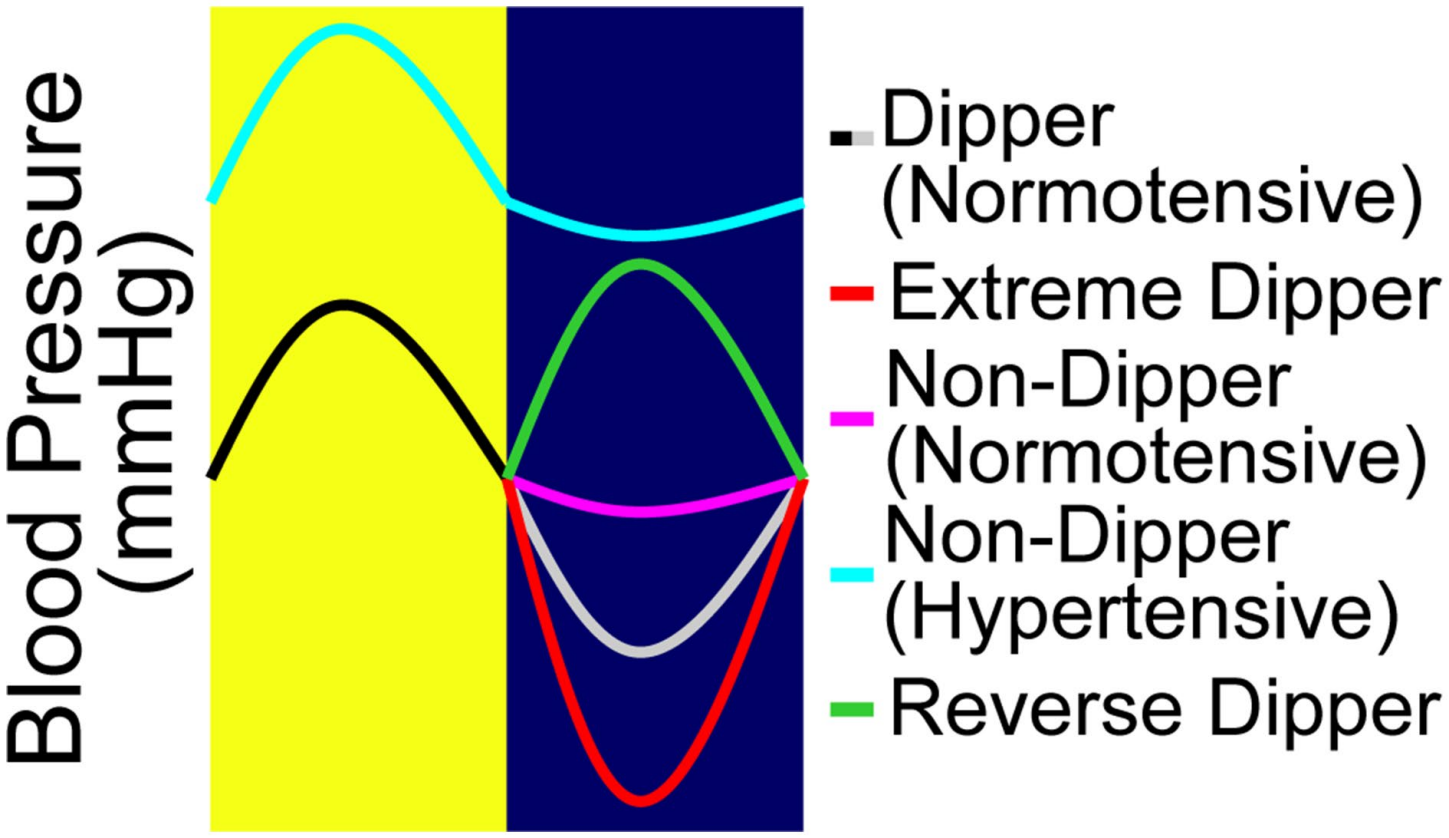
The four nocturnal dipping blood pressure patterns. The fall in blood pressure or the “dip,” is defined as the difference between daytime mean systolic pressure and nighttime mean systolic pressure, expressed as a percentage. There are four different types of blood pressure dipping categories. Blood pressure that dips between 10 and 20% is considered normal dipping (black/gray). Extreme dippers (black/red) dips over >20%. Non-dippers are considered those whose dip less than 10%; they can be characterized as either normotensive non-dippers (black/pink) or hypertensive non-dippers (aqua). Reverse dippers (black/green) are those whose nighttime blood pressure is higher than the daytime blood pressure.

The regulation of blood pressure by the baroreceptors is primarily aimed at inhibiting large variations in blood pressure throughout the day. However, in normal individuals, there is a circadian mediated decrease in blood pressure at nighttime. A term coined “nocturnal blood pressure dipping” has been used to describe the physiologically normal decrease in mean arterial pressure by approximately 10–20% at night (Figure 1; Kastrup et al., 1993). Some reports suggest that this diurnal blood pressure pattern is cardioprotective (Verdecchia et al., 1994). This cardioprotective effect was shown through the administration of blood pressure lowering medications at night provided significantly reduced relative risk of CVD associated events versus those who received their medication in the morning (Hermida et al., 2010). Hypertension is associated with “non-dipping” in which nocturnal blood pressure decreases by <10% at night (Figure 1; O'Brien et al., 1988; Akhtar et al., 2021). Furthermore, individuals with a reverse dipping pattern have also been identified, whose blood pressure is higher at night than during the day (Çoner et al., 2021). This increase in blood pressure during sleep has been shown to be an indicator of developing hypertension. Several studies have shown that the loss of the normal dipping pattern is associated with a significant increase in the risk of developing cardiovascular disease. Ohkubo et al., show that for every 5% loss in nocturnal blood pressure dipping, there is a 20% greater risk for cardiovascular mortality (Ohkubo et al., 2002). Furthermore, a study by Palatini et al. showed that a reduced day-night blood pressure difference and an increased day time BP variability were associated with a higher degree of hypertensive-related cardiovascular complications (Palatini et al., 1992). Moreover, a study by Fagard et al. showed an increased risk for stroke as well associated with hypertension (Fagard et al., 2008). However, a meta-analysis conducted by Maria Gavriilaki et al., concluded that only those individuals expressing a reverse dipping pattern were at a significantly higher risk for a cardiovascular event, whereas the incidence in non-dippers was comparable to normal dippers (Gavriilaki et al., 2020). Although there are some differences between these findings, it is agreed that these irregular dipping patterns have negative implications on long term cardiovascular health outcomes. Interestingly enough, a study showed that individuals with poor quality of sleep were also at an increased risk of expressing non-dipping patterns (Lusardi et al., 1999), showing effects similar to masked hypertension (Hänninen et al., 2013; Tientcheu et al., 2015).

The normal dip in blood pressure is in part regulated by a suppression of the sympathetic nervous system mediated by circadian rhythm (Sherwood et al., 2002). The authors examined the amount of catecholamines, a proxy for sympathetic activity, excreted in urine during the day and at night in patients with normal dipping patterns versus those who were deemed non-dippers. The results showed that although dippers and non-dippers expressed similar rates of catecholamine production during the day, those individuals with non-dipping patterns showed a significantly reduced reduction in catecholamine production at night. These findings indicate that sympathetic output attenuation at night is in part responsible for the dip in blood pressure.

Although the loss of this normal dipping pattern is not entirely understood, it is well accepted that a high salt diet greatly increases an individual’s risk of developing a non-dipping pattern. Hou et al. (2000), investigated the effects of a high salt diet on normotensive and hypertensive individuals and found that this diet attenuated the circadian rhythm of blood pressure in both normotensive and hypertensive salt sensitive individuals (Hou et al., 2000). By measuring plasma norepinephrine concentration (PNE), plasma renin activity (PRA), angiotensin II, aldosterone, erythrocyte sodium content and urinary sodium excretion over a 24-h period, they were able to see how a high salt diet altered the levels of all these blood pressure modulating substances.

Salt-sensitive hypertensive patients tend to exhibit non-dipper-type nocturnal hypertension (Uzu et al., 1997). This is thought to be due to a compensatory mechanism in which the kidneys sense excess salt and attempt to excrete sodium that cannot be fully excreted during the day by raising blood pressure during the night to maintain high renal filtration pressure. In fact, when hydrochlorothiazide 6.25 mg was added to hypertensive patients with inadequate response to 8 mg of candesartan, office blood pressure and nocturnal hypertension improved, and the percentage of non-dipper type patients decreased (Eguchi et al., 2010). Additionally, thiazide diuretics effectively improve nocturnal hypertension, in particular, and normalize the diurnal variation of blood pressure to the dipper type when administered to non-dipper patients (Uzu and Kimura, 1999). However, the effect of normalization of diurnal variation on life expectancy and risk of cardiovascular events is not clear at this time.

Animal studies have greatly contributed to our understanding of the effects of high salt diet on circadian blood pressure disruption. The effects of salt loading on the circadian blood pressure rhythms of Dahl-Salt Sensitive (DSS) rats vs. Dahl-Salt Resistant (DSR) rats was studied by Hashimoto et al. (1994). They showed that DSR rats on a high salt diet showed no difference in mesor (circadian mean) mean arterial pressure (MAP) as compared with DSR rats on a low salt diet. There was, however, a pronounced difference in the MAP of DSS rats on a high vs. low salt diet, seemingly indicating the moderating factor in the disruption of this regular circadian blood pressure cycle to be salt sensitivity (Hashimoto et al., 1994). In Wistar-Kyoto (WKY) and SHR rats, the rhythmic pattern of mean arterial pressure was modified by dietary NaCl in both strains (Fang et al., 2000). Interestingly, plasma sodium and arterial pressure rhythms were inversely correlated in both WKY and SHR. Although plasma sodium rhythms of SHR and WKY were nearly identical, the plasma sodium concentrations were significantly higher throughout the 24-h in SHR. A high salt diet blunted the circadian rhythm of sodium in SHR but not WKY (Fang et al., 2000). A recent study by Sufiun et al. proposes that renal injury may be the cause of the non-dipping pattern (Sufiun et al., 2020). When DSS rats in this study were fed a high salt diet and developed a non-dipper pattern, they appeared to experience renal injury as shown through increased proteinuria. This indicates that a non-dipper pattern may in part be due to renal function deterioration in salt-induced hypertension. Altogether these animal studies strongly suggest that increases in dietic salt may be a contributing factor in the disruption of the circadian rhythm of blood pressure.

Witte et al. examined the relationship between the suprachiasmatic nucleus (SCN) and these dipping patterns in rat models (Witte et al., 1998). Interestingly, their study found that lesioning of the SCN completely removed circadian regulation of blood pressure. To restore circadian blood pressure rhythms along with other cardiovascular parameters, the lesioned rats were then treated with a synthetic melatonin agonist. Despite this, their rhythms did not return, indicating that the SCN plays a key role in maintaining these circadian patterns. In addition to the SCN, there are many other neurogenic modulators of blood pressure. Issues arise when these modulators’ regular functions are disrupted, such as by a high salt diet. Although this exact mechanism by which high salt diet disrupts 24-h blood pressure, has not been fully elucidated, several key factors have been identified. In Huang and Leenen (1994), found that high salt diet greatly increased sympathetic nervous activity (Huang and Leenen, 1994). A buildup of sodium ions within the cerebrospinal fluid activates epithelial sodium channels (ENaCs), increasing the amount of sodium entering the neurons. This high intracellular sodium activates the renin-angiotensin-aldosterone system (RAAS). RAAS activation also causes release of ouabain, an endogenous digitalis like factor (EDLF). These EDLFs have been shown to stimulate sympathetic activity in the brain, leading to an increase in sympathetic outflow to the cardiovascular system, thus, increasing blood pressure. It should be noted that research into EDLFs is ongoing, and their pathways and functions are yet to be fully understood.

Another brain region known to be involved in regulation of blood pressure is the paraventricular nucleus (PVN) of the hypothalamus. In Larson et al. (2017) studied the effects of a high salt diet on the PVNs of Sprague–Dawley rats and found that a high salt diet increased the excitability of PVN neurons (Larson et al., 2017). Their study suggested that the increase in excitability was a result of inhibition of Ca^2+^-ATPase in the endoplasmic reticulum, leading to a depletion of intracellular Ca^2+^. A study by Chapp et al. (2017) expanded on this idea, concluding that increased sodium in the CSF decreased calcium dependent potassium channel currants, promoting PVN cell depolarization and enhanced excitability (Chapp et al., 2017). This increase in excitability of PVN-RVLM neurons proved to be a key factor in sympathoexcitation leading to the development of neurogenic hypertension (Basting et al., 2018). Basting et al. (2018), found that direct stimulation of glutamatergic neurons of the PVN in rat models lead to a direct frequency-dependent rise in blood pressure, while ligation of the PVN-RVLM neurons resulted in a blunted rise in blood pressure following salt treatment (Basting et al., 2018). These results emphasize how constant stimulation of glutamatergic neurons within the PVN by increased CSF sodium concentration can lead to chronic activation and the maintenance of an elevated blood pressure. However, a study by Su et al. (2021) drew a different conclusion regarding the mechanism of how high salt diet alters the function of the PVN (Su et al., 2021). This study suggested that increased reactive oxygen species as well as NLRP3-dependent inflammation caused by high salt concentration in the CSF causes a disruption in the excitatory-inhibitory neurotransmission within the PVN (Su et al., 2021). Although the true physiology may not be clear, these studies all suggest the importance of the PVN on diet-related blood pressure management within the CNS.

A study by Jones et al. (2021) sought to find a connection between the SCN and the PVN, specifically how these two brain regions communicate in order to modulate circadian rhythm (Jones et al., 2021). The authors found a critical connection between these two brain regions in which the SCN released vasoactive intestinal peptide (VIP) onto PVN neurons, stimulating their release of corticotropin-releasing hormone (CRH). Interestingly, they found that this activity peaked around midday, and was followed approximately 3 h later by a peak in calcium activity. They concluded that rhythmic corticosterone release in the body was mediated by this SCN-PVN pathway and played a critical role in the maintenance of circadian rhythm. The complex neural circuits uncovered by these studies may help to elucidate the interplay between circadian rhythms and blood pressure control in the human brain.

Although there are several areas of the brain that have been closely studied for their role in blood pressure regulation, the nTS is one of great importance that has yet to be completely understood. The nTS, being the initial site for the coordination for the baroreflex, is essential in blood pressure regulation (Ciriello et al., 1994). Maintaining a balance of the excitatory and inhibitory neurotransmitters glutamate and GABA within the nTS are key in regulating BP (Sved and Sved, 1989). As such, alterations of the receptors associated with these neurotransmitters have been shown to result in changes in blood pressure (Callera et al., 2000). Studies have highlighted the importance of GABAergic influences within the nTS, showing how introduction of GABA agonists and antagonists can inhibit and enhance the baroreflex, respectively (Brooks et al., 1992; Vitela and Mifflin, 2001). However, the impact of the nTS on blood pressure dipping patterns has yet to be investigated. A study by Affleck et al. (2012) aimed to determine the connection between the nTS and the PVN (Affleck et al., 2012). They utilized neuroanatomical tract tracing and immunohistochemistry to uncover glutamate neurons in the nTS that projected to the PVN in 4 distinct regions, each with phenotypically different neurons populating them. These neurons included pre-sympathetic, GABA, and nNOS containing neurons. With these findings, they hypothesized that the nTS could send excitatory and inhibitory reflex branches to the PVN to mediate cardiovascular homeostasis in response to atrial volume fluctuations. Furthermore, a study by Buijs et al. (2014) used similar techniques to highlight a signaling pathway from the nTS to the SCN (Buijs et al., 2014). This study found that blood pressure elevations not only increased neuronal activity in the nTS, but also in the SCN. Buijs et al. speculated from their findings that the SCN receives blood pressure information directly from the nTS, forming a large circuit in the dynamic response to blood pressure fluctuations. Through these findings, it is evident that these brain regions play a major role in the regulation of blood pressure in response to major fluctuations as well as changes in blood chemistry. Additionally, these regions all interlink to mediate an incredibly complex and poorly understood reflex to alterations in blood pressure.

## Social differences in food availability led to differences in the development of hypertension

Although hypertension affects all types of individuals, there are undeniable health disparities across the United States that puts certain groups of individuals at a greater risk. In a cohort study by Neufcourt et al., they aimed to investigate the independent association of wealth, education, and income with incident hypertension among older adults living in the United States (Neufcourt et al., 2021). Their findings suggest that socioeconomic status, especially wealth, is a strong independent predictor of incident hypertension in older adults. This fact can be attributed to the dietary implications of living in a lower socioeconomic area in which healthy, affordable food options are scarce, otherwise known as “food deserts.” In these areas, large grocery stores are out of walking distance, and driving is not an option for most individuals. For that reason, the easiest food options are small convenience stores that are stocked with unhealthy processed foods that are packed with sodium. These individuals are forced to consume a high salt diet due to their inability to access healthy, natural food options. A study by Ingabire et al. further examined the rates of non-dipping patterns across ethnicities, and found that non-dippers were more likely to be African American, specifically, those with uncontrolled hypertension (Ingabire et al., 2021). Furthermore, African Americans were found to have a higher percentage of patients expressing a non-dipping pattern (Muntner et al., 2015). According to the United States Census Bureau, African American and Hispanic individuals have the highest poverty rate in this country (18.8 and 15.7%, respectively), more than double that of their white and Asian counterparts. All of this information suggests that African American and Hispanic individuals living below the poverty level make up the populations at the greatest risk for developing hypertension secondary to a high salt diet, which further puts them at the greatest risk for severe cardiovascular complications later in life. Furthermore, individuals living in low socioeconomic areas were found to be more reliant on safety net providers rather than primary care physicians offices, likely due to financial insecurities and an inability to afford continuous care (Hussein et al., 2016). This indicates that individuals living in low SES areas are less likely to see a physician to be treated for their hypertension. A study assessing the rates of hypertension awareness, control and treatment across various races showed that African Americans had the lowest percentage of controlled hypertensive patients (Cutler et al., 2008). This lack of access to continuous care puts these individuals at an even greater risk for more dangerous outcomes as their hypertension progresses. A JAMA study by Clark et al. (2019) showed that individuals with hypertension were at a 2 times greater risk of developing CVD compared to non-hypertensive individuals, and that 50% of the African American population was hypertensive by AHA blood pressure guidelines (Clark et al., 2019). Further, this study showed that the population attributable risk for CVD secondary to hypertension was 15% greater in African American adults than in white adults. These results emphasize the increased risk that minority individuals living in low socioeconomic areas have for the development of hypertension, and complications of untreated hypertension.

## Conclusion

Extensive time and research have unveiled a wealth of knowledge regarding the body’s intrinsic mechanisms for blood pressure control. We discussed the body’s first line of surveillance in blood pressure regulation, the baroreceptor reflex, and how its projections to the nTS modulate autonomic output to the heart to compensate for the changes in peripheral pressure. Further, the PVN and SCN are two critical brain regions responsible for blood pressure modulation. We discussed the implications of a high salt diet on the body’s innate abilities to modulate blood pressure, and looked towards recent research that proves how these increased salt concentrations are directly impacting the intrinsic pathways responsible for the maintenance of circadian blood pressure dipping patterns. Lastly, we identify the significant socio-economic imbalances that indirectly increase the risk of CVD amongst underrepresented minorities and low-income communities likely related to structural racism. There are still more questions that need to be answered to fully understand this complex process. Further research should investigate exactly how a high salt diet is correlated to a loss of nocturnal blood pressure dipping, and whether hypertension or loss of blood pressure dipping patterns is the preceding symptom. Specifically, studies to determine the role of high salt diet on modifying the central mechanisms directly related to blood pressure regulation, such as the baroreflex, and how these are reflected in dipping patterns is necessary to develop further therapeutics. Lastly, the root of the high salt diet in our country in relation to food manufacturing processes should also be a topic of discussion.

## Author contributions

DM conceived and designed the study. DM, JV, and DC wrote the first draft of the manuscript. All authors contributed to the article and approved the submitted version.

## Funding

This work was supported by American Heart Association 23AIREA1057412 to DM; NIH LRP 1L40HL165612-01 to DM; and Cooper Biomedical Sciences Internal Competitive Funds to DM. Funding for open access provided by Rowan University Libraries’ Open Access Publishing Fund.

## Conflict of interest

The authors declare that the research was conducted in the absence of any commercial or financial relationships that could be construed as a potential conflict of interest.

## Publisher’s note

All claims expressed in this article are solely those of the authors and do not necessarily represent those of their affiliated organizations, or those of the publisher, the editors and the reviewers. Any product that may be evaluated in this article, or claim that may be made by its manufacturer, is not guaranteed or endorsed by the publisher.
